# Testing the Effectiveness of an Abbreviated Version of the Nutrition Detectives Program

**DOI:** 10.5888/pcd11.130161

**Published:** 2014-04-10

**Authors:** David L. Katz, Judith A. Treu, Rockiy G. Ayettey, Yasemin Kavak, Catherine S. Katz, Valentine Njike

**Affiliations:** Author Affiliations: Judith A. Treu, Rockiy G. Ayettey, Yasemin Kavak, Catherine S. Katz, Valentine Njike, Yale University Prevention Research Center–Griffin Hospital, Derby, Connecticut.

## Abstract

**Introduction:**

Obese or overweight children have an increased risk for chronic diseases. Targeting diet and exercise in schools could help prevent childhood obesity. We have previously shown the effectiveness of a 90-minute nutrition program in improving elementary school students’ food-label literacy. The objective of this study was to investigate the effectiveness of a 45-minute version of the program.

**Methods:**

We conducted a pre–post study in a public school district, with no control group. We provided teacher training and program materials. Participants were 5th-grade students in 5 schools who had parental consent and were willing to take part. We condensed the program to a 45-minute lesson with a presentation and hands-on activity. The lesson showed students why and how to make healthful food choices based on Nutrition Facts panels and ingredient lists. The district’s physical education teachers taught the lesson. The primary outcome measure was food-label literacy (ie, the ability to distinguish between more and less healthful foods using a validated test instrument with Nutrition Facts panels and ingredient lists).

**Results:**

A total of 212 students completed pre–post measures. Following program delivery, we observed a significant gain of 16.2 percentage points in scores overall, ranging from 4.3 percentage points to 23.6 percentage points among schools. Results were similar to those achieved with the 90-minute program.

**Discussion:**

The condensed nutrition program improved students’ food-label literacy while requiring a minimal allocation of time. Further studies in other school districts would be useful.

## Introduction

In 2009–2010, nearly 17% of children and adolescents aged 2 through 19 years were obese, and approximately 32% were either overweight or obese (1). Children who are overweight or obese have an increased risk of developing type 2 diabetes, insulin resistance, heart disease, high blood pressure, metabolic syndrome, and other health-related disorders (2). Childhood obesity tends to track into adulthood (3–5). Thus, the need for obesity prevention among children is essential.

Targeting modifiable risk factors such as diet and exercise early in life is likely to contribute to the prevention of childhood obesity. Schools can become an effective environment for obesity prevention, because no other institution has as much continuous and intensive contact and influence on children during the first 2 decades of life (6). Schools can create an effective environment by promoting good nutrition, physical activity and education, recess, health education, and school health services (7).

Enhancing children’s awareness of and interest in more healthful foods is a potential part of the solution (8–10). One way to enhance awareness and interest is to encourage children to become food-label–literate (11). We developed a 90-minute program, Nutrition Detectives, to help elementary school students develop food-label literacy skills and showed its effectiveness in enhancing students’ ability to distinguish between more and less healthful food products in a variety of food categories (12). Because not all schools have the time or resources to implement the full-length version of this program, our objective was to investigate the effects of offering a condensed version of the program on students’ nutrition knowledge and food-label literacy. We hypothesized that the effect of the condensed version of the program on nutrition knowledge and food label literacy would not differ from that of the original program.

## Methods

### Study design

This was a pre–post study conducted during March through June 2010, after approval by the Griffin Hospital Institutional Review Board (IRB) (IRB study no. 2010/03) by the Yale–Griffin Prevention Research Center (PRC) in collaboration with the New Haven Public Schools (NHPS) District Wellness Committee. The PRC provided teacher training and program materials, developed informed consent materials, and collected pre–post data. The NHPS district was responsible for teaching the program, scheduling data collection, distributing informed consent materials, collecting opt-out forms, and informing the PRC about students who had parental permission to participate in the study. To reduce burden on the district in scheduling data collection, no control schools were used for comparison.

### Study schools

The 5 participating schools were a subset of the district’s elementary/middle schools selected by the NHPS District Wellness Committee and whose principals agreed to participate (Table 1). Of the 5 schools, 4 (Schools A, B, C, and D) were magnet schools with a particular instructional focus, and one (School E) was a traditional school. For the overall student population in each school, the percentages of students ranged from 70.6% to 92.2% for eligibility for free or reduced-price meals, 2.2% to 18.5% for students considered gifted or talented, and 74.9% to 90.8% for minority students The percentage of 5th-grade students in participating schools who met state goals for academic achievement in 2009–2010 ranged from 19.4% to 50.0% for reading, 10.0% to 73.6% for writing, 35.1% to 79.6% for mathematics, and 5.0% to 50.9% for science.

**Table 1:** Demographic Data for Participating Schools in the New Haven Public Schools District, Study on Nutrition Detectives Program, 2010^a^

Characteristic	School A	School B	School C	School D	School E
**Type of school**	Magnet	Magnet	Magnet	Magnet	Traditional
**Instructional focus**	Museums	Arts	Arts	International	Regular
**Grade range**	Pre-K–8	5–8	K–8	Pre-K–8	Pre-K–8
**Overall student population, %**					
Eligible for free or reduced price meals	75.1	81.8	70.6	87.4	92.2
Gifted or talented	3.3	11.0	18.5	2.9	2.2
**Race/ethnicity, %**					
American Indian	0	0	0.2	0.2	0
Asian	1.3	1.7	2.0	2.9	0.6
Black	74.9	49.3	59.7	74.7	91.1
Hispanic	14.6	25.4	13.1	10.1	6.7
White	9.2	23.7	25.1	12.0	1.5
Total minority	90.8	76.3	74.9	88.0	98.5
**Grade 5 academic achievement** b					
Reading	31.4	44.8	50.0	19.4	NA
Writing	41.7	73.6	67.9	36.6	10.0
Mathematics	37.1	63.2	79.6	35.1	NA
Science	27.8	48.4	50.9	19.5	5.0

Abbreviation: NA, not available.

a 2009–2010 school year data from Connecticut State Department of Education Strategic School Profiles (13).

b Percentage of students meeting state goal for Connecticut Mastery Test Scores (ie, more demanding than proficient level but less than advanced level reported in No Child Left Behind Report Cards [14]).

### Study participants

Because the program was incorporated into the health curriculum, all 5th-grade students in participating schools who were present when the Nutrition Detectives lesson was taught took part in the lesson. Study inclusion criteria were 1) being a 5th-grade student in a participating school; 2) having parental consent to complete the quizzes; 3) willingness, availability, and ability to complete the quizzes; and 4) presence at school on the day and in the room where the lesson was taught. Exclusion criteria were students who lacked parental consent, were unwilling/unavailable/unable to complete the quizzes, or were not present for the Nutrition Detectives lesson.

Information sheets and consent forms approved by the Griffin Hospital IRB were sent home to parents or legal guardians of 5th-grade students in study schools. The information sheets provided instructions for parents to opt out of having their child participate by a certain deadline date. All documents and participant information were obtained for research purposes only and strictly maintained to ensure confidentiality. All student assessment data were identified by number, not name.

### Intervention

A few months before this study, the PRC had partnered with the NHPS District Wellness Committee to adapt the 90-minute nutrition program to a 45-minute lesson, to serve as the third of 4 nutrition lessons taught by physical education (PE) teachers in all of the district’s elementary schools. The topics of lessons 1 through 4 were 1) how food affects our bodies; 2) the Food Guide Pyramid; 3) making healthful food choices by using nutrition information on food packages and 5 detective “clues”; and 4) balancing caloric intake and expenditure to maintain a healthy body weight. The nutrition lessons were part of a 5th-grade health curriculum developed by the district and community partners that also included lessons on physical activity, reproductive health, and substance abuse. PRC staff had taught the original 90-minute program to 315 5th-grade students in 8 NHPS schools as part of an unpublished feasibility study (D.L.K. and C.S.K., unpublished data) during the 2007–2008 and 2008–2009 school years and used the same tool to evaluate food label literacy.

The original 90-minute nutrition education program, Nutrition Detectives, (http://www.davidkatzmd.com/nutritiondetectives.aspx), was developed in 2002 and has been periodically updated to reflect changes in food products and refined in content and design according to feedback from teachers and students to strengthen the program’s messages and reduce the potential for misconceptions. Nutrition Detectives conveys the idea of a link between food choices and health, in addition to information on how and what nutritious foods to choose. The key concepts are that “we are what we eat”; students can become “detectives” to learn about the foods they eat; and they can apply 5 “clues” to determine whether food products are healthful (“clued-in”) or less healthful (“clue-less”) choices. The objectives are to enable students to 1) explain why making informed food choices can benefit their health; 2) recognize deception on packages of food products; 3) identify 5 clues to make healthful food choices (Figure); 4) demonstrate the location of the Nutrition Facts panel and ingredient list on food packages; and 5) determine whether foods are healthful choices according to the 5 clues, combined with the Nutrition Facts panels and ingredient lists on their packages. The first half of the program is a PowerPoint presentation designed to engage, motivate, and inform the students. The second half is an activity in which teams of students search through a grocery bag containing food products in a given category, such as cereals, crackers, or snack bars, and decide which products are “clued-in” and which are “clue-less.”

**Figure:**
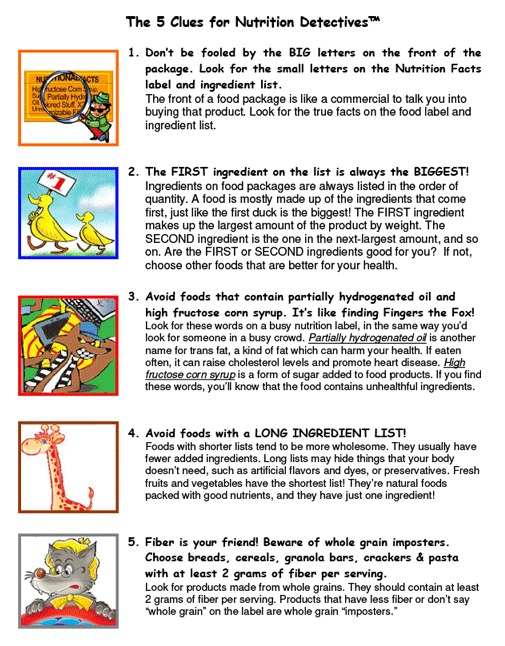
The 5 clues in the school-based Nutrition Detectives program that help children and adolescents to make healthful food choices

The original Nutrition Detectives program included a 96-slide PowerPoint presentation; a 72-page teacher manual with detailed instructions; wall displays; and handouts. The program can be delivered in one 90-minute session, or in 2, 3, or 4 shorter sessions. Nutrition Detectives is freely available to educators; it led to an 18 percentage-point gain in food-label literacy scores (*P* < .01) in the Independence School District in Missouri (12).

The condensed Nutrition Detectives program used in the NHPS study included a 40-slide PowerPoint presentation and a 20-page lesson plan with program objectives, key concepts, vocabulary words, abbreviated instructions, wall displays, and handouts.

In March 2010, PRC staff provided teacher training on the Nutrition Detectives lesson to PE teachers from all K–8 schools in the district as part of a professional development session, including (but not limited to) the schools that elected to participate in the evaluation of the lesson. In May 2010, NHPS PE teachers taught the lesson in 5th-grade classrooms as part of the curriculum.

### Evaluation

The primary outcome measure was food-label literacy and nutrition knowledge on healthful food choices. Children’s ability to choose “better-for-you” foods was assessed by using a previously validated (15) standardized test instrument (Food Label Quiz) that uses Nutrition Facts panels and ingredient lists from 5 categories of food products (breads, crackers, cereals, cereal bars, and cookies) discussed in the Nutrition Detectives program. The quiz consists of 10 questions. Each question asks students to select the more healthful of a given pair of food products on the basis of the nutrition information for each product. The selection of each better-for-you choice is based on one or more of the program’s 5 clues.

PRC research staff administered the Food Label Quiz in participating schools. Pretest and posttest quizzes were scheduled for days and times convenient to school schedules and relative to when the Nutrition Detectives lesson was taught in each school. When the PRC researchers arrived to collect data, the teachers identified the students who would be completing the quizzes. Because of the way informed consent was managed by the schools, the researchers were unable to determine the number of students who lacked parental consent, were unwilling/unavailable/unable to complete the quizzes, or were not present for the Nutrition Detectives lesson (eg, because of absence from school or disciplinary reasons).

Data were entered in an Excel (Microsoft Corporation, Redmond, Washington) spreadsheet and analyzed by using SPSS for Windows version 15.0 (SPSS Inc, Chicago, Illinois). The Food Label Quiz was scored as the percentage of correct responses. Paired *t* tests were used to evaluate the change in the percentage of correct responses of Food Label Quiz scores from pretest to posttest. Student *t* tests were used to assess difference in students’ performance between the 90-minute program and the 45-minute lesson. In all analyses, a 2-tailed α of less than .05 was considered statistically significant. This study was powered at 80% to detect a minimal detectable difference of 18 percentage points between pretest and posttest scores.

## Results

A total of 212 students — 89 boys (42.0% of participants) and 123 girls (58.0%) — completed both the pretest and posttest. There were 26 students (12.3%) from School A, 81 (38.2%) from School B, 49 (23.1%) from School C, 26 (12.3%) from School D, and 30 (14.1%) from School E. Mean baseline quiz scores ranged from 40.0% in School A to 59.0% in School C (Table 2). After delivery of the Nutrition Detectives lesson, a significant improvement overall (all 5 schools combined) of 16.2 percentage points was observed in students’ scores (*P* < .001) on the quiz between pretest and posttest measures (Table 2). Improvements in scores were observed in all 5 schools, ranging from 4.3 percentage points to 23.6 percentage points. These improvements were significant in School B, School C, and School D. Overall, boys’ scores improved by 12.1 percentage points, and girls’ scores improved by 19.1 percentage points. Girls’ scores improved significantly more than boys’ scores (*P* = .04).

**Table 2:** Nutrition Detectives Food Label Quiz Scores, New Haven Public Schools District, 2010^a^

Variable	No. of Quiz Scores	Pretest Score, % (SD)	Posttest Score, % (SD)	Change, Percentage Point (SD)	*P* Value^b^
**Overall**	212	52.5 (20.5)	68.6 (20.3)	16.2 (24.3)	<.001
**Sex**					
Boys	89	52.8 (21.9)	64.9 (21.9)	12.1 (26.3)	.009
Girls	123	52.2 (19.4)	71.3 (18.8)	19.1 (22.4)	<.001
**School**					
A (n = 26)	26	40.0 (15.0)	47.7 (14.5)	7.7 (23.0)	.10
B (n = 81)	81	53.8 (20.0)	77.4 (15.4)	23.6 (20.8)	<.001
C (n = 49)	49	59.0 (20.7)	76.1 (15.4)	17.1 (22.6)	<.001
D (n = 26)	26	53.1 (20.9)	66.5 (20.4)	13.5 (29.0)	.03
E (n = 30)	30	48.7 (20.6)	53.0 (21.5)	4.3 (26.6)	.38

a The Food Label Quiz (13) was scored as the percentage of correct responses to 10 questions.

b *P* values determined from paired student *t* test.

## Discussion

The overall results of this study were similar to those from the unpublished feasibility study of the 90-minute program in which students scored an overall 52.6% (SD, 19.0%) in the pretest (vs 52.5% for the 45-minute program; *P* = .92) and 67.6% (SD, 23.7%) in the posttest (vs 68.6% for the 45-minute program; *P* = .65). The students in both studies also showed similar gains in food-label literacy scores (15.0 percentage points for the 90-minute program and 16.2 percentage points for the 45-minute lesson; *P* = .57). The overall results are also similar to the 18.1 percentage-point pretest–posttest gain in scores shown in the evaluation of the 90-minute Nutrition Detectives program in the Independence School District using the same assessment tool (12). The comparable gain in scores in this study was achieved despite edits required to condense the program materials into a 45-minute lesson, which included removing much of an original interactive discussion intended to motivate students to change their eating habits and removing several slides that reinforced the program’s messages. These edits apparently did not diminish the program’s effect on food-label literacy.

Although NHPS students in this study experienced an overall 16.2 percentage-point gain in food-label literacy scores after the 45-minute lesson, the extent of improvement varied among schools. The differences in scores at baseline or the gain in scores after the Nutrition Detectives lesson could partly be attributed to differences among schools in 1) overall instructional focus (ie, among magnet schools or between magnet and traditional schools; 2) students’ socioeconomic status; 3) percentage of gifted or talented students 4); percentage of minority students; or 5) overall level of student academic achievement. For example, the 2 schools with the highest baseline food-label literacy scores and the greatest gain in scores (School B and School C) were magnet schools with the highest proportions of gifted and talented students, the lowest proportions of minority students, and the highest proportions of students meeting state goals for academic achievement. In contrast, the 2 schools with the lowest baseline scores and small nonsignificant gains in scores (School A and School E) had lower proportions of gifted and talented students, the highest proportions of minority students, and lower proportions of students meeting state academic achievement goals. An exception was School D, which had a significant gain in food-label literacy scores but had a low proportion of gifted and talented students, a high proportion of minority students, and a low proportion of students meeting state academic achievement goals.

Other possible explanations for differences in scores include differences in the lesson delivery setting, PE teachers’ styles of teaching, and students’ degree of readiness to learn the lesson. For example, in one school (School A) with only a small gain in scores, the PE teacher taught the Nutrition Detectives lesson in the gymnasium rather than the classroom and combined students from all classrooms for the lesson; in addition, student disciplinary issues led to postponement of the lesson until a day when students were more cooperative.

Overall, the improvement in food-label literacy scores after the lesson was greater among girls (19.1 percentage points) than among boys (12.1 percentage points). However, this is not surprising given that throughout elementary, middle, and high school, girls tend to earn higher grades than boys in all major subjects (16).

Our study suggests that schools can take a lead in offering food literacy education that will enable children to identify more healthful food choices at school, at home, and in other settings. If the Nutrition Detectives program is used to achieve this goal, it appears that either the original 90-minute program or the condensed 45-minute version of the program could be used with similar success. Whereas the 90-minute version is already designed to fit well into school curricula without requiring a great deal of time, the 45-minute version could provide an additional option for schools with a crowded curricula.

This study has several limitations. The extent to which the results can be generalized can be questioned because the study group was limited to 1 school district and to schools within the district that chose to participate. The self-selection of schools to participate may have been due to a higher level of motivation to offer and assess the effects of nutrition education, which could bias the results toward the null. There was no control group for comparison in evaluating the 45-minute lesson. The differences in score improvements among schools could be attributed to differences in instructional focus, demographic makeup of students, academic achievement levels of students, or inconsistencies in program delivery among schools.

Despite these study limitations, the 45-minute Nutrition Detectives program appears to be an effective means of teaching elementary school students to identify healthful food choices while requiring minimal time in the school curriculum. Compared with the full-length Nutrition Detectives program, it requires less time and fewer resources to implement. Other schools can likely use this program to achieve similarly positive results, although the program may require academic readiness for students to best comprehend and apply the program’s content. Further confirmation of the condensed program’s effectiveness, either alone or in combination with other health programs, could be obtained through a randomized controlled trial with other school populations.
